# Determining the Molecular Background of Endometrial Receptivity in Adenomyosis

**DOI:** 10.3390/biom10091311

**Published:** 2020-09-11

**Authors:** Erika Prašnikar, Tanja Kunej, Katja Repnik, Uroš Potočnik, Jure Knez, Borut Kovačič

**Affiliations:** 1 Department of Reproductive Medicine and Gynaecological Endocrinology, University Medical Centre Maribor, 2000 Maribor, Slovenia; prasnikare@gmail.com (E.P.); borut.kov@ukc-mb.si (B.K.); 2 Department of Animal Science, Biotechnical Faculty, University of Ljubljana, 1230 Domžale, Slovenia; tanja.kunej@bf.uni-lj.si; 3 Centre for Human Molecular Genetics and Pharmacogenomics, Medical faculty, University of Maribor, 2000 Maribor, Slovenia; katja.repnik82@gmail.com (K.R.); uros.potocnik@um.si (U.P.); 4 Laboratory for Biochemistry, Molecular Biology and Genomics, Faculty of Chemistry and Chemical Engineering, University of Maribor, 2000 Maribor, Slovenia; 5 Department of Gynaecologic and Breast Oncology, University Medical Centre Maribor, 2000 Maribor, Slovenia

**Keywords:** adenomyosis, candidate genes, endometrial receptivity, endometriosis, gene expression, gene set enrichment analysis (GSEA), multi-omics, protein–protein interaction network (PPIN)

## Abstract

Background: Adenomyosis is a gynaecological condition with limited evidence of negative impact to endometrial receptivity. It is commonly associated with endometriosis, which has been shown to alter endometrial expression patterns. Therefore, the candidate genes identified in endometriosis could serve as a source to study endometrial function in adenomyosis. Methods: Transcripts/proteins associated with endometrial receptivity in women with adenomyosis or endometriosis and healthy women were obtained from publications and their nomenclature was adopted according to the HUGO Gene Nomenclature Committee (HGNC). Retrieved genes were analysed for enriched pathways using Cytoscape/Search Tool for the Retrieval of Interacting Genes/Proteins (STRING) and Reactome tools to prioritise candidates for endometrial receptivity. These were used for validation on women with (*n* = 9) and without (*n* = 13) adenomyosis. Results: Functional enrichment analysis of 173, 42 and 151 genes associated with endometriosis, adenomyosis and healthy women, respectively, revealed signalling by interleukins and interleukin-4 and interleukin-13 signalling pathways, from which annotated *LIF*, *JUNB*, *IL6*, *FOS*, *IL10* and *SOCS3* were prioritised. Selected genes showed downregulated expression levels in adenomyosis compared to the control group, but without statistical significance. Conclusion: This is the first integrative study providing putative candidate genes and pathways characterising endometrial receptivity in women with adenomyosis in comparison to healthy women and women with endometriosis.

## 1. Introduction

Embryo implantation, characterised by synchronised interaction between vital embryo and maternal receptive endometrium [1], is restricted to the short time window of implantation (WOI), which appears in the mid-secretory (MS) phase of the menstrual cycle [2]. Endometrial-associated factor [3] could have a role in repeated embryo implantation failure (RIF), which prolongs assisted reproductive technique (ART) treatment, causing psychological trauma to infertile couples [4]. Therefore, omics technologies have been applied to identify potential biomarkers of uterine receptivity, which could contribute to tailored infertility treatment [3]. Based on the knowledge from omics studies, the first clinical test for endometrial receptivity, the so-called “Endometrial receptivity array” (ERA, Igenomix, Spain), was developed. The ERA test is designed to evaluate a personalised WOI on a basis of transcriptomic signature of endometrial biopsy. Since WOI could be displaced during the secretory (S) phase in some infertile women, the ERA test tends to determine optimal timing of embryo transfer in ART treatments [5]. 

Adenomyosis is a gynaecological pathology where tissue similar to endometrium (ectopic endometrium) is located within the myometrium, the smooth musculature of the uterus [6]. Advances in high-resolution transvaginal ultrasound (TVUS) and magnetic resonance imaging (MRI) contribute to the common detection of adenomyosis in infertility diagnosis [7]. No exact criteria for sonographic features of adenomyosis are established [6], so the prevalence in a population of infertile patients is not exactly known. Puente et al. [8] diagnosed adenomyosis in 24.4% (*n* = 248/1015) and Hashim et al. [9] in 7.5% (*n* = 24/320) of women seeking ART treatment. Salim et al. [10] associated adenomyosis with a negative impact on endometrial receptivity when women underwent the first cycle of ART treatment. Lower pregnancy (22.2% vs. 47.2%) and higher miscarriage (50.0% vs. 2.8%) rates were observed in women with diagnosed adenomyosis compared to women exhibiting anovulatory menstrual cycles, endometriosis, or unexplained, tubal or male factor subfertility [10]. Higher rates of RIF (34.7% vs. 24.4%) between women with and without adenomyosis were also observed by Puente et al. [8]. In addition, a 28% decrease in the probability of clinical pregnancy in women with vs. without adenomyosis was predicted with the use of meta-analysis [11]. On the other hand, the molecular mechanism of hampered endometrial receptivity in adenomyosis is not well understood and mainly based on candidate gene/protein designed studies [12,13].

Adenomyosis is classified as “Endometriosis of uterus” according to the International Statistical Classification of Diseases and Related Health Problems 10th Revision (ICD-10) World Health Organisation (WHO) version for 2019 (https://icd.who.int/browse10/2019/en). Adenomyosis commonly coexists with endometriosis [14], which is characterised by ectopic endometrium located outside of the uterine cavity. The prevalence of endometriosis in infertile women with normospermic partners is estimated to be 47% (*n* = 104/221) [15], and it has been shown to alter the molecular patterns of endometrial receptivity. Numerous differentially expressed transcripts and proteins were identified using genome-wide profiling of endometrial samples during windows of receptivity in women with and without endometriosis [16,17,18,19,20]. Therefore, the reported altered endometrial signatures from pathophysiologically similar endometriosis provide a source of candidate genes to study receptivity in adenomyosis. 

Biological network maps and functional enrichment analyses enable prioritisation of candidate genes for validation experiments in target tissue [21,22]. The network-based approach provides insight into the global molecular organisation of studied biological components (protein-coding or functional RNAs, proteins and metabolites). Nodes of the network present biological components, while edges present their physical relationships (protein–protein or RNA–DNA interactions, regulatory relationships between genes and transcription factors, binding, activation, inhibition, etc.) [21]. In the functional enrichment-based approach, a list of biological components is analysed by bioinformatics tools that use biological knowledge base, statistical testing, mathematical analyses and computational algorithms to recognise their relationship patterns [22]. In the present study, candidate genes for endometrial expression validation in adenomyosis were prioritised on the basis of gene set enrichment analysis (GSEA) using reported molecular signatures characterising endometrial receptivity.

Therefore, the aims of the present study were to (1) screen for reported transcripts/proteins associated with endometrial receptivity in adenomyosis, endometriosis and healthy women; (2) edit the nomenclature of extracted transcripts/proteins to develop gene lists specific for adenomyosis, endometriosis and healthy groups; (3) obtain enriched pathways associated with retrieved genes using bioinformatics network and functional enrichment-based approaches; (4) prioritise candidate genes for validation experiment; and (5) analyse expression patterns of selected genes in endometrial biopsy samples collected during expected windows of receptivity in women with and without adenomyosis.

## 2. Materials and Methods

**Scheme 1:**
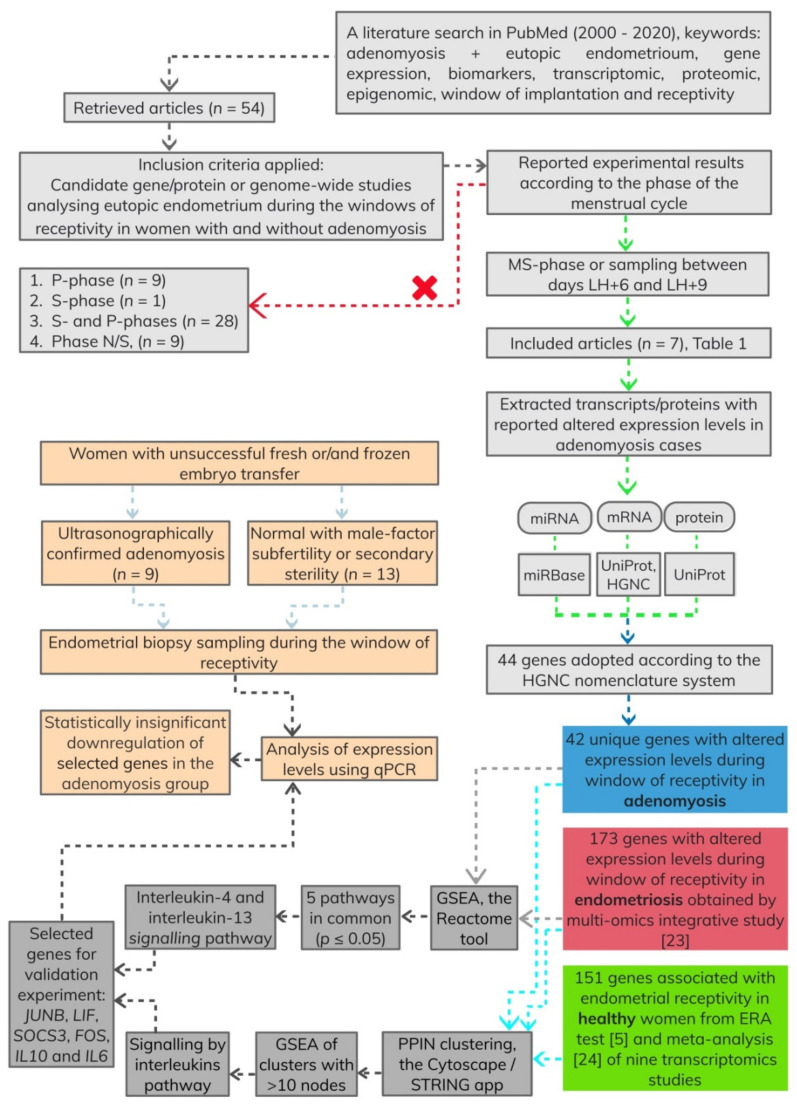
Workflow and main results of the study. Main steps included literature mining, bioinformatics prioritisation of candidate genes and validation expression analysis of endometrial biopsy samples from women with and without adenomyosis. Legend: GSEA = gene set enrichment analysis, ERA = endometrial receptivity array, HGNC = Hugo Gene Nomenclature Committee, STRING = Search Tool for the Retrieval of Interacting Genes/Protein, P = the proliferative phase of the menstrual cycle, S = the secretory phase, MS = the mid-secretory phase, N/S = not specified, LH = luteinising hormone. LH+6 and LH+9 refer to the WOI which is between six and nine days after LH surge.

The workflow of the study is presented in Scheme 1.

### 2.1. Identification of Relevant Studies for Development of Gene List

The development of the gene list associated with altered endometrial molecular patterns in adenomyosis was based on a literature search conducted in the PubMed literature database. Combinations of the keywords “adenomyosis” and “eutopic endometrium”, “gene expression,” “proteomic,” “transcriptomic,” “epigenomic,” “window of implantation,” “receptivity,” and “biomarkers” were used. The search was conducted from 2000 until January 2020. 

The gene list associated with endometrial receptivity in endometriosis was adopted from our previous integrative study of published genome-wide studies [23]. Briefly, top reported transcripts (protein-coding and functional RNAs) and proteins with altered expression patterns in the endometria of women with endometriosis were synthesised in the gene catalogue. Retrieved genes were further sorted according to the phases of the menstrual cycle. For the present analysis, genes from the MS-phase were extracted [23].

Gene sets characterising endometrial receptivity in healthy women were provided from two publications based on transcriptomics experiments: 238 genes from a genomic diagnostic ERA test [5] and 57 genes from a meta-analysis of nine transcriptomics studies [24]. 

The nomenclature of extracted transcripts and proteins from the literature survey was adopted according to the HUGO Gene Nomenclature Committee (HGNC) database (last updated 4 May 2020) [25] which is assigned to provide official symbols for human genes. Editing of the nomenclature across studies enabled downstream bioinformatics analysis for prioritisation of candidate genes for the validation experiment. 

### 2.2. GSEA Using Retrieved Genes Associated with Adenomyosis, Endometriosis and Healthy Endometrium 

The interaction of all retrieved genes was determined in the global network approach and was further clustered to distinguish molecular patterns in women with uterine disorders (endometriosis and adenomyosis) compared to healthy women. Search Tool for the Retrieval of Interacting Genes/Proteins (STRING) is a database which enables assessment and integration of protein–protein interactions of submitted biological components, such as genes [26]. The developed gene lists associated with endometrial receptivity in adenomyosis, endometriosis and healthy groups were projected into the protein–protein interaction network (PPIN) using the Cytoscape 3.8.0. software platform [27,28] and STRING app. The ClusterMaker2 app was further applied for identification of clusters with tightly connected proteins within the obtained network. The Markov cluster (MCL) algorithm was set to 2.5 for inflation value and the overall STRING confidence score was used as the array source. Obtained clusters with more than 10 nodes were analysed using STRING functional enrichment analysis (or GSEA). Obtained pathways with a false discovery rate (*FDR*) of ≤0.05 were considered for statistical significance. Annotated nodes to the top eight overrepresented terms of Gene Ontology (GO), Kyoto Encyclopedia of Genes and Genomes (KEGG) and Reactome pathways were visualised using a split donut chart after redundant terms were removed using STRING app filtering. 

### 2.3. GSEA Using Retrieved Genes Associated with Adenomyosis and Endometriosis Endometrium

Molecular knowledge from well-studied endometriosis was used as a model to identify putative pathways characterising endometrial receptivity in poorly studied adenomyosis. The GSEA in the Reactome bioinformatics tool version 72, released on 16 March 2020 [29] was applied for overlapping enriched pathways between retrieved genes associated with adenomyosis and endometriosis. Reactome is a database that provides a pathway over-representation (enrichment) analysis of submitted genes. The tool also provides a graphical map where known biological processes and pathways in human biology are visualised in hierarchical order [29]. Obtained enriched pathways with statistical significance (*p* ≤ 0.05) were considered for overlap between the two gene lists. 

### 2.4. Participating Women

The experimental protocol was approved by the National Ethics Committee of the Republic of Slovenia (0120-259/2018/16) and the study was conducted in accordance with the Helsinki Declaration of 175, as revised in 2013. Written informed consent was obtained from every participant prior to inclusion in the study. Participating women with unsuccessful fresh or/and frozen embryo transfer during ART treatment were recruited from the Department of Reproductive Medicine and Gynaecological Endocrinology, University Medical Centre Maribor, Maribor, Slovenia. 

Women with sonographic markers of adenomyosis according to a TVUS diagnostic infertility workup were recruited for the adenomyosis group. Women with normal TVUS findings and no history of adenomyosis, myoma, endometriosis or polycystic ovary syndrome (PCOS) who underwent ART due to male factor of subfertility or expose secondary sterility were recruited for the control group. An additional TVUS examination using Voluson E8 (GE Healthcare Austria GmbH & Co OG, Zipf, Austria) of the uterus and pelvis in all participating women was performed on the day of eutopic endometrium biopsy sample collection. Sonographic criteria for adenomyosis diagnosis were asymmetrical myometrial thickening not caused by the presence of fibroids, linear striations, parallel shadowing, myometrial cysts, hyperechoic islands and/or the presence of adenomyoma [30]. 

### 2.5. Clinical Data

Clinical data of age, body mass index (BMI), sterility (primary = never pregnant, secondary = achieved pregnancy), number of ART treatments before enrolling in the study and male factor subfertility based on evaluated spermatozoa concentration and morphology according to the WHO Laboratory Manual for the Examination and Processing of Human Semen, fifth edition [31] were obtained from the electronic medical records stored in our Meditex IVF database (Critex GmbH, Regensburg, Germany). Endometrial thickness was determined by TVUS examination prior to endometrial biopsy. 

### 2.6. Endometrial Biopsy Sample Collection

Endometrial biopsy samples were obtained from the uterine cavity during the natural menstrual cycle using a Probet endometrial suction curette (Gynetics Medical Products N.V., Lommmel, Belgium). The luteinising hormone (LH) peak was determined by urinary LH ovulation rapid test cassettes (Hangzhou AllTest Biotech Co., Ltd, Hangzhou, P.R. China). Sampling was performed between days LH+6 and LH+9 (LH = 0 is the day of LH surge) corresponding to expected WOI. Two anovulatory women, one in the adenomyosis and second in the control group, received human chorionic gonadotropin (hCG) (Ovitrelle, Merck Europe B.V., Amsterdam, The Netherlands) to trigger ovulation. Sampling was calculated according to the day of hCG administration (hCG = 0) on day hCG+6 or hCG+7. The endometrial biopsy was immediately placed in RNAlater solution (catalog number AM7021, Thermo Fisher Scientific Baltics UAB, Vilnius, Lithuania) and stored overnight at +4 °C, then transferred to −80 °C until total RNA isolation. 

### 2.7. Total RNA Isolation

Whole endometrial tissue sample was used for total RNA isolation using the miRNeasy Mini Kit (catalog number 217004, Qiagen GmbH, Hilden, Germany) according to the protocol recommended by the manufacturer. The kit also enables isolation of miRNA fraction, but that was not used in this study. Concentration and purity for each RNA sample were determined spectrophotometrically using Synergy2 (BioTek Instruments, Winooski, VT, USA). The RNA integrity number (RIN) for each sample was assessed by RNA Nano 6000 Assay Kit (catalog number 5067-1511, Agilent Technologies, Waldbronn, Germany) on the 2100 Bioanalyzer System (Agilent Technologies, Waldbronn, Germany). 

### 2.8. Gene Expression Analysis by Quantitative PCR (qPCR)

Total RNA was first reverse-transcribed to 1 µg cDNA using a SuperScript IV Vilo Master Mix (catalog number 11756050, Thermo Fisher Scientific Baltics UAB, Vilnius, Lithuania) according to the manufacturer’s instructions. Selected mRNAs, with corresponding hydrolysis probe IDs (catalog number 4331182, Life Technologies Corporation, Pleasanton, CA, USA) were: *FOS* (Hs99999140_m1), *LIF* (Hs01055668_m1), *JUNB* (Hs00357891_s1), *SOCS3* (Hs01000485_g1), *IL6* (Hs00174131_m1) and *IL10* (Hs00961622_m1). Glycealdehyde-3-phosphate dehydrogenase (*GAPDH*; Hs02786624_g1) and 18S ribosomal RNA (*18S* rRNA; Hs99999901_s1) were used as reference genes. The final qPCR reaction mixture with 10 µL volume was as follows: 5 µL of 2× diluted TaqMan Gene Expression Master Mix (catalog number 4369016, Thermo Fisher Scientific Baltics UAB, Vilnius, Lithuania), 0.5 µL of 20× diluted selected hydrolysis probe assay, 2.5 µL of nuclease-free water and 2 µl of 10× diluted cDNA template. In each run, RNase-free water without cDNA (no-template controls) and mRNA added in reverse transcription reaction mix without reverse transcriptase (no reverse transcription controls) were included as negative controls. Quantification was performed by a LightCycler480 instrument (Roche, Basel, Switzerland). The qPCR protocol was as follows: uracil-N-glycosylase (UNG) incubation at 50 °C for 2 min and polymerase activation at 95 °C for 10 min, followed by 45 cycles of denaturation at 95 °C for 15 s then annealing and extension at 60 °C for 1 min. Each sample was analysed in duplicate and average quantification cycle (Cq) values were used for calculations. 

Final results were presented as fold differences in gene expression relative to the normalised calibrator, calculated by the *2^−ΔΔCq^* method [32]. For each studied sample, the geometrical mean of *GAPDH* and *18S* rRNA (*Cq_sample,R_*) was used for normalisation and Δ*Cq* values were further calculated as follows: Δ*Cq_sample_ = Cq_sample_,_gene of interest_ − Cq_sample,R_*. The value of calibrator (Δ*Cq_calibrator_*) for each gene of interest was determined as the average of retrieved Δ*Cq_sample_* values from all samples investigated with qPCR, including case and control study groups, and was used in the following formula: ΔΔ*Cq_sample_ =* Δ*Cq_sample_ −* Δ*Cq_calibrator_*. 

### 2.9. Statistical Analysis 

Age, body mass index (BMI), endometrial thickness, number of previous ART treatments and fold change of gene expression levels are presented as medians with 95% confidence interval (CI). Non-parametric Mann–Whitney testing using SPSS 25.0 software (IBM Corporation, Armonk, NY, USA) was applied to compare study groups for statistically significant differences. The significance level was equal to ≤0.05. 

## 3. Results

**Figure 1:**
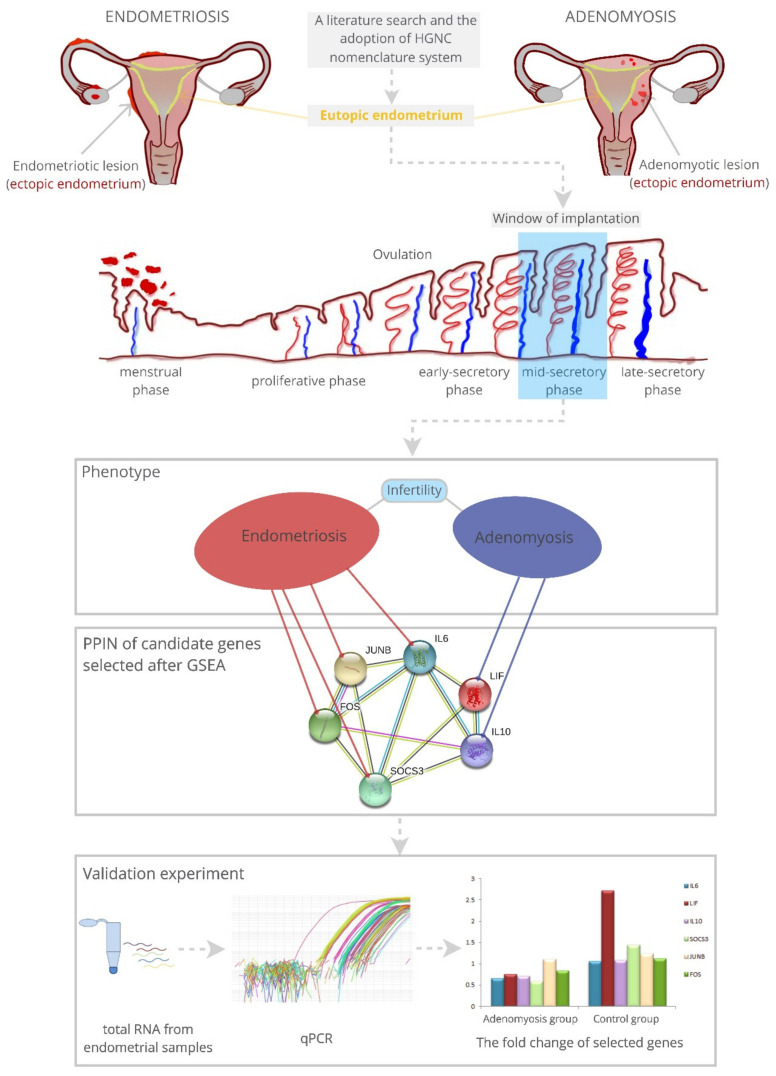
Graphical abstract of the study. Literature screening and HGNC nomenclature provided genes associated with endometrial receptivity that were further analysed for enriched pathways. Six candidate genes for experimental validation in adenomyosis were prioritised. Two selected genes originated from the adenomyosis group of 42 genes, and four from the endometriosis group of 173 genes. Protein–protein interaction network (PPIN) of selected genes is visualised using STRING database. Candidate genes were further validated in endometrial biopsy samples collected during window of receptivity in women with and without adenomyosis. Legend: GSEA = gene set enrichment analysis, STRING = Search Tool for the Retrieval of Interacting Genes/Protein. PPIN = Protein–protein interaction network, HGNC = Hugo Gene Nomenclature Committee.

The workflow of the literature mining and the main results are presented in Scheme 1. Bioinformatics prioritisation and validation of candidate genes is visualised in graphical abstract in Figure 1. The literature search and HGNC nomenclature provided genes associated with endometrial receptivity in adenomyosis, endometriosis and healthy women. These were further used for functional enrichment analysis to prioritise candidate genes for the validation experiment. Selected genes were analysed for expression levels in endometrial biopsy samples collected during the expected window of receptivity in women with and without adenomyosis who underwent ART treatment.

### 3.1. Developed Gene Lists 

The literature search associated with adenomyosis provided 54 studies, further divided according to the phase of the menstrual cycle (Scheme 1). Articles with candidate protein/gene and genome-wide study designs that analysed eutopic endometrium in women with and without adenomyosis during expected endometrial receptivity (LH timed to the WOI or MS-phase of the menstrual cycle) were further considered. Among 54 retrieved studies, 47 were excluded from the present analysis because they were performed in S or proliferative (P) phase, or the phase of the menstrual cycle was not specified. Among the 54 studies, six candidate gene/protein [12,13,33,34,35,36] and 1 genome-wide study [37] were included in gene list development. Extracted transcripts (mRNAs and ncRNAs) and proteins reported to be differentially expressed in adenomyosis cases are listed in Table 1, and their corresponding official humane gene symbols were provided according to the HGNC database. Of the total 44 retrieved genes, two (*LIF* and *HOXA10*) were repeated among studies. The final gene list included 42 unique genes associated with endometrial receptivity in adenomyosis. In addition, Table 1 provides information regarding composition and sample size of adenomyosis and control groups and indication of endometrial samples collection.

The gene list associated with endometrial receptivity in endometriosis included 173 unique genes obtained from our previous multi-omics analysis [23]. Retrieved genes originated from six genome-wide studies, including 1 at the proteomics level [38], three at the transcriptomics level [16,17,18], one at the transcriptomics and ncRNomics level [19] and one at the epigenomics level, providing differentially expressed genes associated with altered methylation level [20].

The list associated with endometrial receptivity in healthy women included 151 unique genes. From the meta-analysis [24], 39 genes were extracted because they were experimentally validated and confirmed to exhibit altered expression patterns in endometrial samples LH+8 compared to LH+2. From the ERA test [5], 143 genes were extracted because they were validated by RT-PCR, included in a test as gold standard biomarkers of receptivity, or belonged to the endometrial receptivity transcriptomic signature. Of the total 182 genes, 31 overlapped between studies.

The retrieved 42 genes associated with endometrial receptivity in women with adenomyosis (A = 42), 173 in women with endometriosis (E = 173) and 151 in healthy women (H = 151) are listed in Table 2. 

**Figure 2:**
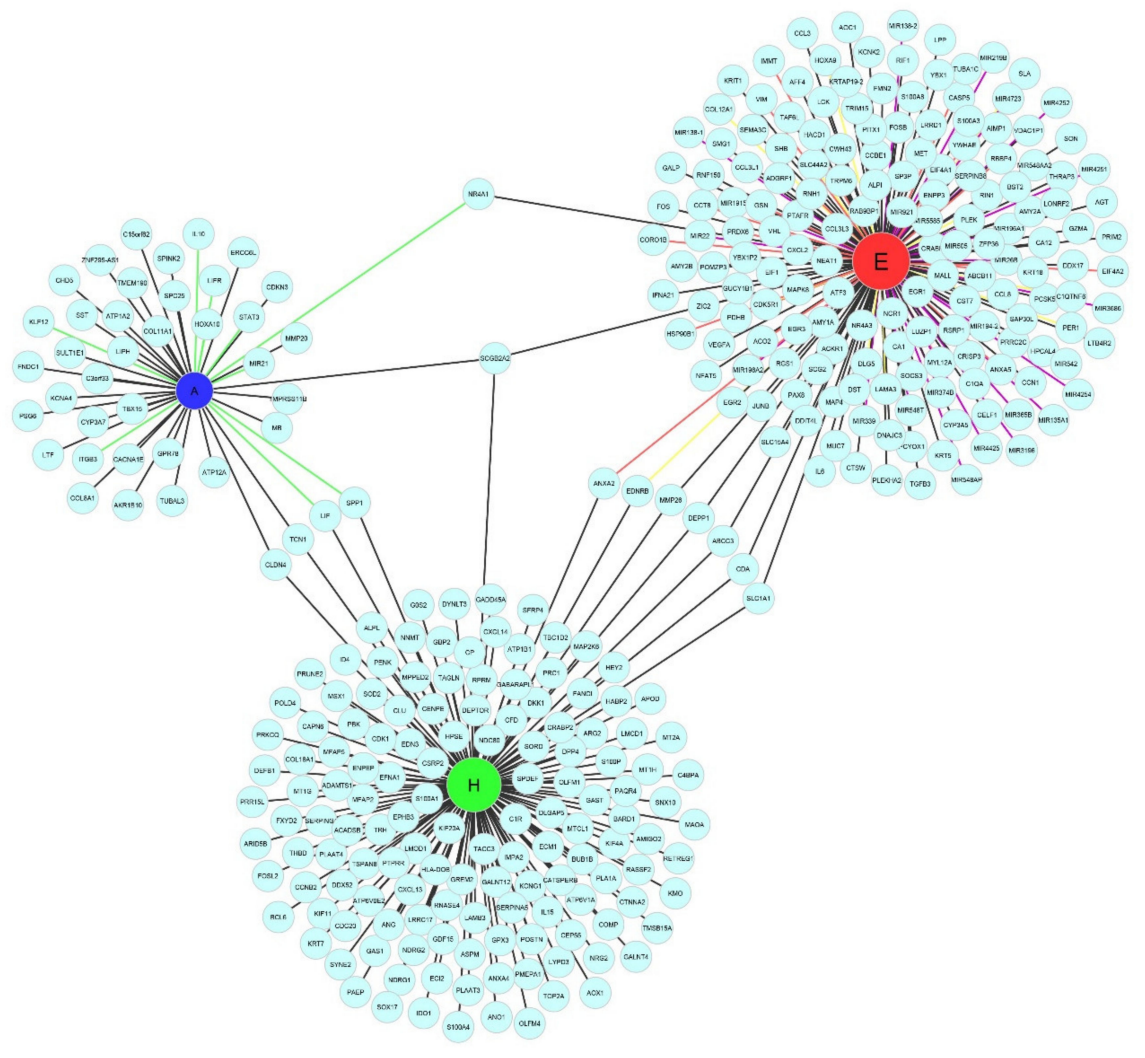
Retrieved genes associated with endometrial receptivity in women with adenomyosis (A = 42) or endometriosis (E = 173) and healthy women (H = 151) and overlap across groups. Colour of edges between genes represents the type of study design/omics level from which genes with reported molecular dysregulation were retrieved through literature mining: green, candidate protein/gene study design; black, transcriptomics study design; yellow, proteomics study design; red, epigenomics study design; and violet, ncRNomics study design.

Figure 2 visualises these genes in adenomyosis, endometriosis and healthy groups connected with edges presenting the study design/omics level that was applied in the source publications that reported altered endometrial expression levels of associated genes. *SPP1*, *LIF*, *TCN1* and *CLDN4* were common to adenomyosis and healthy groups of genes, while *ANXA2*, *EDNR8*, *MMP26*, *DEPP1*, *ABCC3*, *CDA* and *SLC1A1* were common to endometriosis and healthy groups. *NR4A1* repeated in adenomyosis and endometriosis, while *SCGB2A2* was on all three genes lists. 

### 3.2. Enriched Pathways after PPIN Clustering 

**Figure 3:**
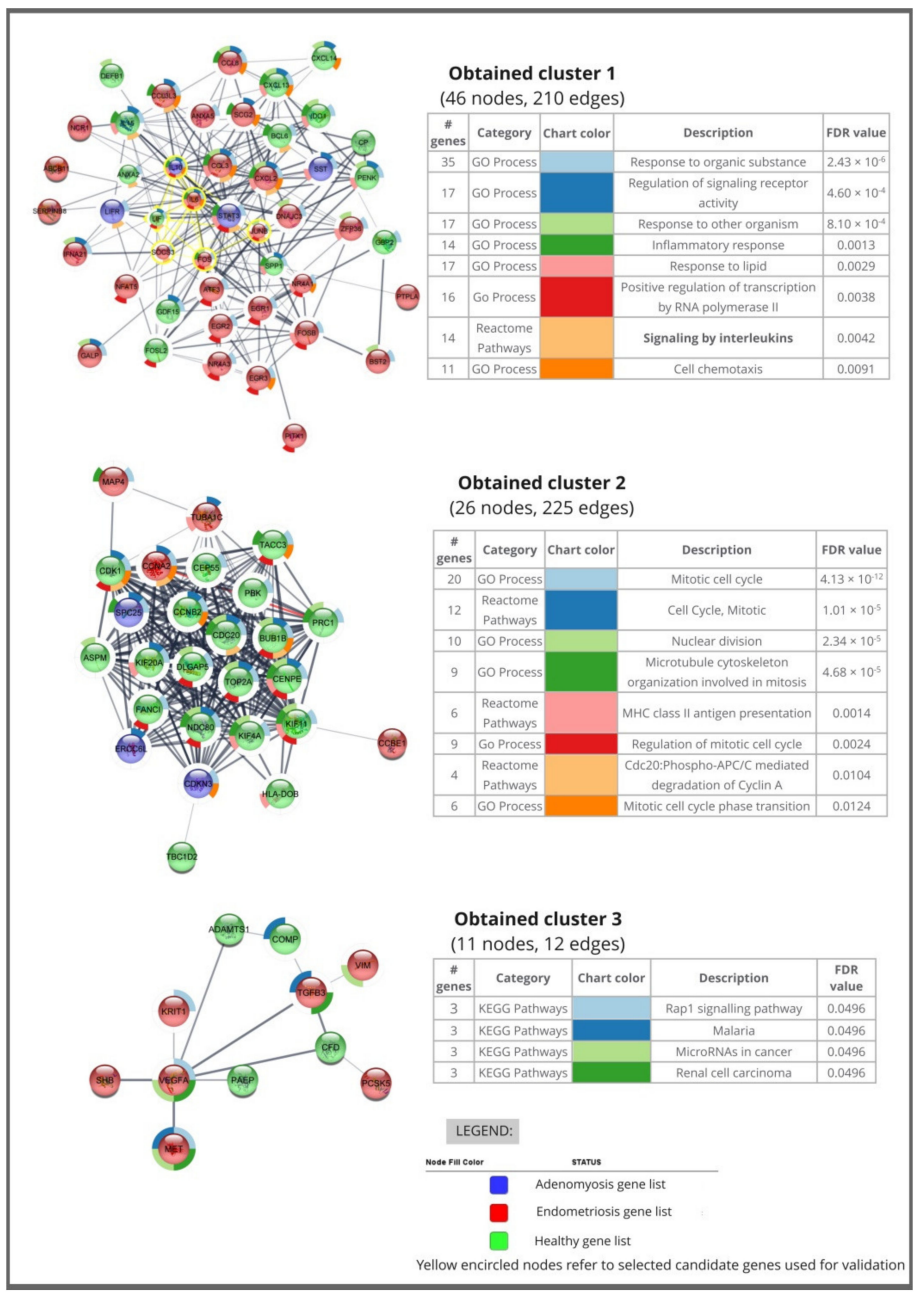
Clusters with more than 10 nodes obtained from PPIN associated with endometrial receptivity in women with adenomyosis or endometriosis and healthy women. GSEA was applied for enriched pathways and annotated nodes were coloured in split donut charts. Signalling by interleukins pathway (marked in bold) from cluster 1 served as a source for prioritisation of genes for validation experiment. Complete PPIN is provided in Supplementary Figure S1. STRING database projected retrieved gene symbols *ADGRF1*, *HACD1*, *GUCY1B1*, *CCN1*, *TMSB15A* and *RETREG1* in the PPIN as GPR110, PTPLA, GUCY1B3, CCNA2, TMSNB and FAM134B, respectively.

Projection of all retrieved genes resulted in a PPIN with 315 nodes and 1130 edges. Network clustering (workflow presented in Supplementary Figure S1) resulted in three clusters containing more than 10 nodes, named cluster 1, cluster 2 and cluster 3. Figure 3 presents pathways associated with nodes in clusters 1, 2 and 3 after removal of redundant enriched terms. Cluster 1 included 210 edges and 46 nodes that were mapped to all three gene lists: adenomyosis (A = 4), endometriosis (E = 28) and healthy (H = 14). Functional enrichment analysis of cluster 1 provided 102 enriched terms of GO processes, KEGG and Reactome pathways, including response to organic substance, regulation of signalling receptor activity, inflammatory response, signalling by interleukins and cell chemotaxis. Cluster 2 included 26 nodes which were mainly mapped to the healthy gene list (H = 19) and was connected with 225 edges. Three and four nodes were mapped to adenomyosis (A = 3) and endometriosis (E = 4) gene lists, respectively. Enrichment functional analysis of cluster 2 provided 54 enriched terms of GO processes and Reactome pathways. Most enriched terms were associated with cell cycle and mitosis, including mitotic cell cycle, nuclear division, MHC class II antigen presentation and regulation of mitotic cell cycle. Cluster 3 was constructed from 11 nodes, mapped to endometriosis (E = 7) and healthy (H = 4) gene lists, and connected with 12 edges. Four enriched KEGG pathways were associated with cluster 3, including Rap1 signalling pathway and MicroRNAs in cancer. Complete GSEA results of all three clusters are presented in Supplementary Table S1. 

### 3.3. Overlapping Enriched Pathways between Adenomyosis and Endometriosis Gene Lists 

GSEA was applied to identify overlapping pathways between genes associated with endometrial receptivity in adenomyosis and endometriosis. Altogether, 56 enriched pathways associated with endometriosis and 45 with adenomyosis were provided, which are listed in Supplementary Table S2. Table 3 lists five overlapping pathways: gene and protein expression by JAK-STAT signalling after interleukin-12 stimulation, interleukin-10 signalling, interleukin-4 and interleukin-13 signalling, neutrophil degranulation and PTK6 activation of STAT3, together with annotated genes, *p*-value and *FDR*.

### 3.4. Prioritisation of Candidate Genes for Validation Experiment

Candidate genes were prioritised from the obtained enriched pathways with a similar biological role. Signalling by interleukins (R-HSA-449147) associated with cluster 1 (annotated as *LIF, LIFR, STAT3, IL15, JUNB, FOS, SOCS3, ANXA2, BCL6, IL6, IL10, CXCL2, CCL3* and *CCL3L3*) and interleukin-4 and interleukin-13 signalling (R-HSA-6785807) enriched by both adenomyosis and endometriosis gene lists (annotated as *IL10, STAT3, LIF, SOCS3, IL6, VIM, FOS, JUNB, VEGFA* and *HSP90B1*) were highlighted as source pathways for candidate genes selection. According to the Reactome pathway database, selected pathways are hierarchically sorted within immune response. Six annotated genes were selected for validation analysis: LIF interleukin 6 family cytokine (*LIF*) and interleukin 10 (*IL10*), originating from the adenomyosis gene list, and JunB proto-oncogene, AP-1 transcription factor subunit (*JUNB*), interleukin 6 (*IL6*), Fos proto-oncogene, AP-1 transcription factor subunit (*FOS*) and suppressor of cytokine signalling 3 (*SOCS3*) from the endometriosis gene list (Figure 1).

### 3.5. Patients

A total of 22 women between 29 and 42 years old involved in ART treatment were classified to the study groups: ultrasonographically confirmed adenomyosis group (*n* = 9) and control group with male factor subfertility or secondary sterility (*n* = 13). The most frequently observed sonographic finding in adenomyosis cases was asymmetrical myometrial thickening (88.9% of cases). One adenomyosis patient was diagnosed with coexisting myoma. Two women in the control group were diagnosed with small myoma (7 mm) or bilateral hydrosalpinx. No differences between the composition of study groups were observed, except that endometrial thickness was statistically significantly lower in the adenomyosis group that the control group (7.3 vs. 9.2 mm (*p* = 0.030)). Demographic and clinical characteristics of the study groups are presented in Table 4. 

### 3.6. Expression Patterns of Candidate Genes

**Figure 4:**
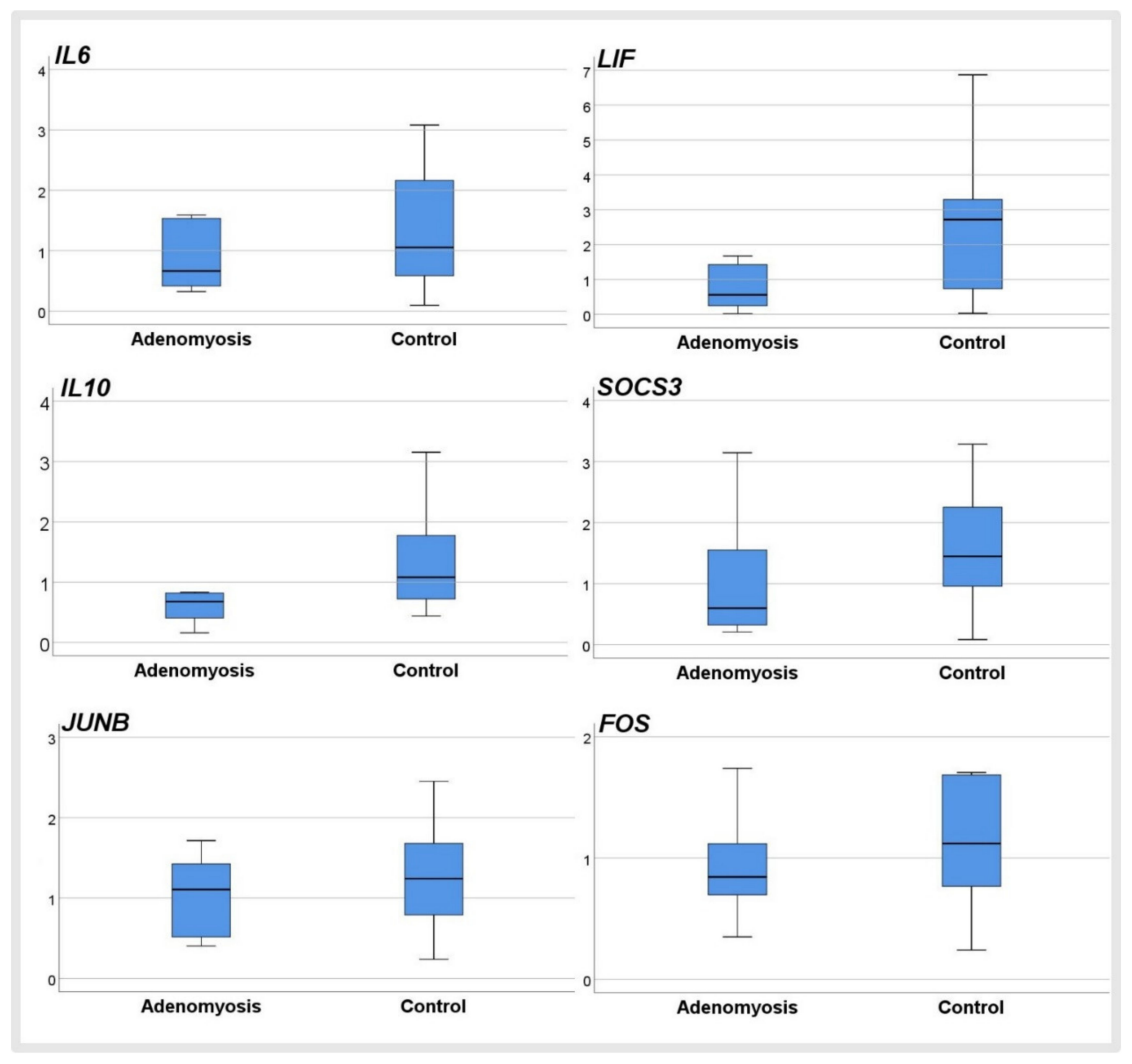
Relative expression levels of selected candidate genes in adenomyosis and control study groups. Selected genes were normalised to expression level of *GAPDH* and *18S* rRNA reference genes.

Isolated total RNA from endometrial biopsy samples collected during the window of receptivity were used to verify whether selected candidate genes *LIF*, *SOCS3*, *FOS*, *JUNB*, *IL6* and *IL10* were differentially expressed between adenomyosis and control groups. The A_260_/A_280_ ratios and RIN values of all RNA samples were above 2.0 and >8, therefore the samples were suitable for further expression analysis with qPCR. No statistically significant differences in gene expression levels between study groups were observed. However, median values (Table 5) indicate downregulation of selected genes in the adenomyosis group. Expression patterns of selected genes relative to normalised calibrator in adenomyosis and control groups are presented in Figure 4. Gene expression levels between study groups remained statistically insignificant when women with coexisting uterine pathologies from case and control groups were excluded from the calculation (data not shown). 

## 4. Discussion

In the present study, prioritisation of candidate genes for an endometrial validation experiment in poorly studied adenomyosis was based on bioinformatics integration of published endometrial expression signatures associated with endometrial receptivity (Scheme 1). A literature survey and the HGNC nomenclature system provided 42 genes associated with endometrial receptivity in adenomyosis. In addition, 173 and 151 genes, respectively, were obtained from well-studied endometriosis patients and healthy women. General and more specific putative dysregulated endometrial pathways in women with adenomyosis were identified using retrieved genes and GSEA applied in Cytoscape/STRING app and Reactome tool. Six candidate genes annotated in selected pathways of immune cytokine signalling were further experimentally validated for expression patterns during the receptivity window in women with and without adenomyosis who underwent ART treatment. 

### 4.1. Integration of Reported Signatures and Highlighted Enriched Pathways

Endometrium is highly active tissue under the control of fluctuating steroid sex hormones which enable tissue growth, shedding and regeneration in a cyclic manner [39]. The knowledge of biological events contributing to the ability of endometrium to become receptive to an embryo is limited, with an overlap of simultaneously reported up- and downregulated genes across transcriptomics studies performed in healthy women [24], and those coexisting uterine pathologies, such as endometriosis [2,23] and adenomyosis [40]. 

In the present study, we attempted to identify additional genes with altered expression patterns in women with adenomyosis based on published data regarding endometrial receptivity. Therefore, retrieved genes associated with reported altered molecular expression patterns during the window of endometrial receptivity in women with adenomyosis and endometriosis were integrated in the PPIN context together with biomarkers of uterine receptivity assumed to characterise healthy women. Further PPIN clustering and downstream GSEA performed by the Cytoscape/STRING app provided the signalling by interleukins pathway within cluster 1 (Figure 3). Annotated nodes in this pathway originated from gene lists associated with adenomyosis, endometriosis and healthy women as well. This could mean that the signalling by interleukins characterising endometrial receptivity in healthy women might be dysregulated in women with endometriosis and adenomyosis. A more specific putative pathway regarding dysregulated interleukin signalling was identified based on overlapping enriched pathways between 42 and 173 genes associated with adenomyosis and better-studied endometriosis, respectively. In that way, the overlapping interleukin-4 and interleukin-13 signalling pathway was identified after GSEA was applied in the Reactome tool (Table 3). 

According to the Reactome pathway database, enriched signalling by interleukins and interleukin-4 and interleukin-13 signalling pathways exposing similar biological role, i.e., immune cytokine signalling. Chaouat et al. pointed out the important role of immune cytokines in embryo implantation [41]. According to the literature, regulation of the local immune response protects the embryo from maternal immunity. During the S-phase, the decidua stimulates an influx of diverse immune cell populations in the endometrium tissue, including uterine natural killer (uNK) cells, lymphocytes and macrophages [39,42]. IL-4 and IL-13, together with IL-5, IL-9 and IL-10, belong to the type 2 cytokines, which are secreted from helper T (Th) lymphocytes. In general, these interleukins stimulate embryo implantation and trophoblast invasion, therefore, assumed to be protective for pregnancy [43]. Based on highlighted enriched pathways from the present GSEA, it was hypothesised that dysregulated patterns of affected endometrial receptivity in women with adenomyosis are associated with protective interleukins for embryo implantation. 

Only published genome-wide studies were considered for present integrative analysis of genes associated with endometrial receptivity in women with endometriosis and healthy women. However, Enciso et al. [44] suggested an additional panel of 40 candidate genes as putative biomarkers for rapid determination of uterine receptivity status by qPCR. Authors discussed that only 7 out of 40 genes is in common with the ERA test [44]. To additionally verify the biomarker potential of those 40 candidate genes we performed pathway enrichment analysis using the Reactome tool. The top retrieved enriched pathway was interleukin-4 and interleukin-13 signalling with *p* = 4.38 × 10^−9^ and *FDR* = 1.26 × 10^−6^, which is in the accordance with our bioinformatics results. The interleukin-4 and interleukin-13 signalling contains 211 entities taking part in 46 reactions, according to the Reactome pathway database, meaning it could present a great pool of candidate genes for endometrial receptivity. Despite *LIF*, *IL6*, *IL10*, *JUNB*, *FOS* and *SOCS3* showing no statistically altered expression patterns between study groups, their lower expression levels were observed in the adenomyosis group. Therefore, *STAT3*, *VIM*, *VEGFA* and *HSP90B1* that were also annotated in interleukin-4 and interleukin-13 signalling in the present GSEA need to be validated to verify potential downregulation of this pathway in women with adenomyosis.

Candidate genes of the present expression analysis were prioritised on the basis of enrichment analysis using genetic loci that were gathered from different omics levels. However, limited correlation between transcriptomics and proteomics levels has been observed previously. Namely, Vogel and Marcotte [45] discussed that there is only 40% correlation between relative mRNA abundances and corresponding protein concentration in mammals as a consequence of post-transcriptional, translation and protein degradation regulation [45]. Therefore, in the future studies it is necessary to extend the research to regulatory mechanisms occurring downstream synthesised mRNA. 

### 4.2. The Role of Selected Genes in Reproductive Biology

Prioritised candidate genes of the present study have been widely studied in the field of reproductive biology. LIF is a glycoprotein cytokine which enhances decidualisation in humans and mice. It is considered as a biomarker of endometrial receptivity [46]. When human endometrial Ishikawa cells and epithelial ECC-1 cells from endometrial adenocarcinoma were treated with LIF, higher expression levels of *ITGAV*, *ITGB3* and *ITGB5* adhesion molecules were observed; these are required for attachment of the embryo trophoblast to the receptive endometrial surface [47]. Serafini et al. reported a 6.4-fold higher chance of pregnancy in women with stronger immunohistochemical staining for LIF in secretory endometrium biopsy samples prior to ART treatment [48]. Downregulation of endometrial *LIF* mRNA and protein observed in women with adenomyosis [12,13] and unexplained infertility [49] was associated with affected receptivity. 

IL-10 is an anti-inflammatory cytokine which acts as a negative regulator of macrophage and T lymphocyte cell activation [50]. It is upregulated during early pregnancy to maintain maternal immunotolerance for the embryo [51]. Wang et al. reported downregulation of secreted IL-10 during endometrial receptivity in adenomyosis [35]. 

IL-6 is a pro- and anti-inflammatory cytokine involved in acute phase response, B cell maturation, macrophages and type 1/2 Th cell differentiation [52]. In regulatory menstruating women, expression of endometrial IL-6 is assumed to be low in the P- and ES-phases; then, it gradually rises in the MS-phase and reaches peak expression in the LS-phase [53]. Higher levels of endometrial IL-6 during the WOI in controlled ovarian stimulation (COS) cycles were observed in women with adenomyosis [54]. In addition, upregulation of *IL6* was observed in primary culture of endometrial stromal cells (ESCs) from women with adenomyosis after co-culture with macrophages. The findings were further associated with a potential gain of proliferative ability of ectopic endometrial implants [55]. On the other hand, its downregulation in the LS-phase was associated with its role in endometriosis pathogenesis [56]. 

FOS and JUNB are subunits which dimerise to form a transcriptomic factor complex, activator protein 1 (AP-1). AP-1 regulates the expression of downstream genes with roles in cell cycle regulation, including proliferation, differentiation, apoptosis and response to stress [57]. Raimundo et al. associated downregulation of the FOS-JUNB pathway with altered differentiation of smooth muscle progenitor cells and development of myomas [58]. Baiyong et al. demonstrated that dysregulation of JUNB can turn on the differentiation of Th lymphocyte populations. Naive CD4 T lymphocytes were first isolated from JUNB-positive transgenic mice and further differentiated in the Th1 cell population. However, overexpression of JUNB prioritised the synthesis of cytokine IL-4, which is normally exclusive to Th2 population cells [59]. In women with endometriosis, upregulation of *JUNB* mRNA [18,60], *FOS* mRNA [18] and FOS protein [61] in secretory endometrium was reported. On the other hand, Morsch et al. observed no difference in phosphorylated levels of FOS when comparing women with and without endometriosis [62]. 

SOCS3 belongs to the family of intracellular proteins that supress cytokine signalling and have a role in negative regulation of inflammatory response [63]. Dong et al. suggested that reduced SOCS3 signalling may increase inflammatory response in placental trophoblasts leading to preeclampsia. Overexpression of SOCS3 in placental JEG-3 cell culture caused enhanced secretion of pregnancy-protective IL-10 [64]. Braunschweig et al. demonstrated that silencing of *SOCS3* enhanced cytotoxicity or killing activity in NK-92 cell culture, which resembles NK cells of decidua [65]. 

### 4.3. Endometrial Tissue Variability

The difference in expression patterns between study groups was not statistically significant in the present study which might be due to whole-tissue samples used for analysis. Suhorutshenko et al. [66] demonstrated that expression contribution of low abundant cell types in heterogeneous tissue could be masked by more abundant types. They compared paired endometrium biopsy samples collected in pre-receptive (early-secretory, ES) and receptive (mid-secretory, MS) states and estimated that ES samples consisted of 65% stromal and 35% epithelial cells, while the proportion of stromal and epithelial cells in MS samples was 46% and 54%, respectively. Further computational adjustment of RNA sequencing data according to obtained proportions of cell types (deconvolution) identified non statistically significant expression for approximately 74% of a total 3591 differentially expressed transcripts that were retrieved without deconvolution, indicating on stromal and epithelial cells unique gene expression profiles [66]. Additional molecular studies, such as in situ hybridisation (ISH) or immunohistochemistry (IHC) staining are needed to localise potential expression patterns of selected candidate genes or corresponding proteins specific to endometrial cell types or cellular compartments. 

Only patients treated for infertility were considered eligible for the present study. To exclude for possible confounding factors and minimise the influence of other gynaecological pathologies affecting the endometrium, only couples with male factor subfertility were included in the control group. Our study shows that endometrial expression pattern could be disrupted during WOI in women with adenomyosis and endometriosis compared to healthy controls. This could suggest lower endometrial receptivity and embryo implantation rates in this group of women; however, this would need to be proved in future clinical trials.

Finally, observed statistically non-significant gene expression differences between study groups could also be due to the personal biological variations since small sample size in both groups was used. Additional studies with larger sample size should address these limitations to determine whether selected candidate genes are differentially expressed in women with adenomyosis. 

### 4.4. Limitations of the Study

Despite extensive literature search and bioinformatics analysis our study has some limitations. 1. Limited published data and heterogeneity of studies. Genes used in the present integrative study were retrieved from the literature survey, focusing on heterogeneous studies with poor overlap of identified molecular signatures characterising normal or affected endometrial receptivity. 2. Prioritisation of selected genes. In the present expression analysis six candidate genes were selected, however, *STAT3*, *VIM*, *VEGFA* and *HSP90B1* annotated in the interleukin-4 and interleukin-13 signalling or *LIFR, STAT3, IL15, ANXA2, BCL6, CXCL2, CCL3* and *CCL3L3* annotated in the signalling by interleukins were not used for validation. 3. The present study included small sample size in both adenomyosis and control groups. Retrieval of endometrial samples is long-lasting process since strict inclusion criteria.

### 4.5. Future Directions

This was a preliminary study intended to show altered endometrial expression patterns in women with adenomyosis. As the sampling and processing of endometrial biopsies were performed in the same manner and isolated RNA samples exhibited high RIN values, genome-wide profiling could provide novel loci specific for adenomyosis. Understanding the molecular background of endometrial receptivity could help determine the impact of diagnosed adenomyosis on the fertility capacity of affected women seeking ART treatment. 

## 5. Conclusions

The diagnosis of adenomyosis has been negatively associated with implantation rate, but this is supported by little molecular evidence of affected endometrial receptivity. Based on data integration, extensive bioinformatics analysis and preliminary validation experiment, our study contributes toward understanding of molecular background characterising endometrial receptivity in adenomyosis. Genes and pathways identified in the present enrichment analysis are a source of stronger candidate genes for further validation analysis regarding endometrial receptivity. Identifying and understanding endometrial molecular organisation could contribute to the development of new concepts for personalised endometrial preparation before embryo transfer. 

## Figures and Tables

**Table 1:** Retrieved studies reporting altered expression levels of transcripts/proteins in endometrial samples during frame of receptivity in women with adenomyosis. Nomenclature (gene symbol and approved gene name) of extracted data was edited according to the HUGO Gene Nomenclature Committee (HGNC) database. Letters A and C refer to adenomyosis and control women/group, respectively. Symbols ↑ and ↓ refer to reported up- and down-regulated, respectively, expression level of genes and proteins when compared adenomyosis with the control group in the source reference.

**Study Design of Retrieved Studies**	**Biotype of Assessed Biological Entity in Source Reference**	**Biological Entities Assessed in Source Reference**	**The HGNC Gene Symbol**	**Approved Gene Name**	**Reported Up-(↑) or Down-(↓) Regulation in Adenomyosis (A) Compared to Control (C) Group, (Statistical Significance)**	**Indication and Procedure for Endometrial Tissue Samples Collection**	**Inclusion Criteria and Composition of A. Number (*n*) of Participants**	**Inclusion Criteria and Composition of C. *n* of Participants.**	**Source Reference**
**Candidate gene/protein**	miRNA	miR-21	*MIR21*	microRNA 21	~50% ↓ (*p* < 0.001)	Endometrial biopsy between days 19 and 23 of the menstrual cycle	Confirmed 2 of 5 sonographic features of *A.* Aged 31.4 ± 0.7 years. *n* = 12	≥1 normal pregnancy and/or delivery. Aged 30.4 ± 0.6 years. *n* = 12	[36]
	protein	KLF12	*KLF12*	Kruppel like factor 12	~2-fold ↑ (*p* < 0.001)				
	protein	NR4A1	*NR4A1*	Nuclear receptor subfamily 4 group A member 1	~50% ↓ (*p* < 0.001)				
	protein	IL-10	*IL10*	Interleukin 10	40% ↓ (*p* < 0.001)	Endometrial biopsy in LH+7 using an endometrial curette	>2 of 5 sonographic features of *A*, clinical symptoms (secondary and progressive dysmenorrhea, menorrhagia and menostaxis), and clinical signs (homogenous enlargement of local uplift of the uterus, firmness and tenderness). Aged 40 years or younger. *n* = 23	Tubal factor infertility. Aged 40 years or younger. *n* = 23	[35]
	protein	HOXA10	*HOXA10*	Homeobox A10	~50% ↓ (*p* < 0.001)				
	Protein phosphorylation	p(Y705)-STAT3	*STAT3*	Signal transducer and activator of transcription 3	~50% ↓ (*p* < 0.001)				
	mRNA, protein	LIFR	*LIFR*	LIF receptor subunit alpha	0.3-fold ↓ mRNA (*p* < 0.01). Protein ↓ according to H-score: - glandular cells 43.6 in A vs. 92.3 in C (*p* < 0.001). - stromal cells: 27.3 in A vs. 57.7 in C (*p* < 0.001).	Laparoscopically assisted vaginal hysterectomy (LAVH) or myomectomy (part of C samples) between days 19 and 23 of the menstrual cycle.	Diagnosed *A* preoperatively with clinical symptoms and ultrasonogram. A confirmed postoperatively by pathologist. Aged 39.8 ± 0.8 years. - Diffuse A, *n* = 5. - Focal A, *n* = 4.	- Intramural or subserosal leiomyoma, *n* = 16. - Submucosal leiomyoma, *n* = 3. Aged 41.2 ± 0.5 years.	[13]
	mRNA, protein	LIF	*LIF*	LIF interleukin 6 family cytokine	0.15-fold ↓ mRNA (*p* < 0.01). protein ↓ according to the H-score: - glandular cells 61.5 in A vs. 231.8 in C (*p* < 0.001). - stromal cells: 40.7 in A vs. 135.4 in C (*p* < 0.001).				
	mRNA, protein	Integrin β3	*ITGB3*	Integrin subunit beta 3	mRNA ↓: Median value 6.2 in A vs. 21.5 in C (*p* < 0.01). protein ↓ according to the H-score: 2.0 in A vs. 2.7 in C (*p* < 0.05).	A group: hysterectomy for dysmenorrhea, hysteroscopy and diagnostic curettage for history of infertility. C group: testing for tubal patency or hysterectomy for pathological changes of the cervix. Ultrasound monitoring of menstrual cycle for endometrial sampling between days 7 and 9 following ovulation. Histologically confirmed mid-late secretory phase.	Enlarged uterus revealed by MRI and/or high level of serum CA125. Aged 40.1 ± 2.7 years. - Dysmenorrhea, *n* = 11. - History of infertility, *n* = 17.	Born at least 1 child and used some form of contraception with: - tubal factor of infertility or - pathological changes of the cervix. Aged 40.9 ± 2.1 years. *n* = 27	[34]
	mRNA, protein	OPN	*SPP1*	Secreted phosphoprotein 1	mRNA ↓: Median value 12.2 in A vs. 24.2 in C (*p* < 0.01). protein ↓ according to the H-score: 2.1 in A vs. 2.7 in C (*p* < 0.05).				
	mRNA, protein	LIF	*LIF*	LIF interleukin 6 family cytokine	Protein ↓ according to the H-score: 2.0 in A vs. 2.7 in C (*p* < 0.05).	Laparoscopy for tubal ligation, testing for tubal patency, or women without endometriosis who had hysterectomy for pathological changes of the cervix. Endometrial sampling by curettage and dating determined by histology.	Preoperative *A* diagnosis on clinical presentation, enlarged uterus determined by TVUS and/or high values of CA125. A confirmed by two histopathologists. Aged 40.3 ± 2.7 years. Total *n* = 28 with: - infertility. *n* = 21 or/and - dysmenorrhea. *n* = 11.	Fertile with: - tubal ligation - pathologic changes of the cervix Aged 40.9 ± 2.6 years. *n* = 27	[12]
	protein	HOXA10	*HOXA10*	Homeobox A10	↓ according to the H-score: 1.4 in A vs. 2.1 in C (*p* < 0.001).	A group: hysterectomy for hypermenorrhea or adnexal mass. *A* confirmed by histopathology. C group: hysterectomy for pelvic organ prolapse or benign adnexal mass. Histologically confirmed MS-phase of the menstrual cycle	Aged between 25 to 52 years old. *n* = 19	Fertile with: - pelvic organ prolapse or, - benign adnexal mass. Aged between 25 to 52 years old. Total *n* = 12	[33]
**Genome-wide**	mRNA	TMPRSS11B	*TMPRSS11B*	Transmembrane serine protease 11B	215.5 fold change (FC) ↑, (pfp* = 0.0421)	Systematic TVUS to detect ovulation. When a follicle of 17–18 mm was detected, LH peak was determined by urinary test. Endometrial biopsies sampling in LH+7 using Pipelle catheter.	A diagnosed by MRI and TVUS - Never pregnant, *n* = 2 - Two term pregnancies, *n* = 1 - A single pregnancy, *n* = 1 - Two early miscarriages, *n* = 1 - A miscarriage, *n* = 1	Young women with regular menses, no uterine or endocrine anomalies, proven fertility (previous spontaneous pregnancy at term). Normal results in a uterine ultrasonography. *n* = 6	[37]
	mRNA	CHD5	*CHD5*	Chromodomain helicase DNA binding protein 5	31.8 FC ↑ (0.00000) Confirmed by qPCR validation				
	mRNA	SST	*SST*	Somatostatin	10.6 FC ↑ (0.0350) Confirmed by qPCR validation				
	mRNA	SPBC25	*SPC25*	SPC25 component of NDC80 kinetochore complex	9.8 FC ↑ (0.0367)				
	mRNA	FLJ20105	*ERCC6L*	ERCC excision repair 6 like, spindle assembly checkpoint helicase	9.1 FC ↑ (0.0496)				
	mRNA	AKR1B10	*AKR1B10*	Aldo-keto reductase family 1 member B10	8.7 FC ↑ (0.0317) Confirmed by qPCR validation				
	mRNA	CDKN3	*CDKN3*	Cyclin dependent kinase inhibitor 3	8.0 FC ↑ (0.0406)				
	mRNA	ATP1A2	*ATP1A2*	ATPase Na+/K+ transporting subunit alpha 2	6.2 FC ↑ (0.04069 Confirmed by qPCR validation				
	mRNA	MB	*MB*	Myoglobin	5.8 FC ↑ (0.0262)				
	mRNA	KCNA4	*KCNA4*	Potassium voltage-gated channel subfamily A member 4	5.5 FC ↑ (0.0255)				
	mRNA	MMP20	*MMP20*	Matrix metallopeptidase 20	5.1 FC ↑ (0.0468)				
	mRNA	FNDC1	*FNDC1*	Fibronectin type III domain containing 1	5.0 FC ↑ (0.0464)				
	mRNA	TUBAL3	*TUBAL3*	Tubulin alpha like 3	4.9 FC ↑ (0.0425)				
	mRNA	SPINK2	*SPINK2*	Serine peptidase inhibitor Kazal type 2	4.8 FC ↑ (0.0406)				
	mRNA	COL11A1	*COL11A1*	Collagen type XI alpha 1 chain	4.6 FC ↑ (0.0493)				
	mRNA	LOC220115	*/*		4.1 FC ↑ (0.0278)				
	mRNA	LIPH	*LIPH*	Lipase H	3.9 FC ↑ (0.0356)				
	lncRNA	C21orf121	*ZNF295-AS1*	ZNF295 antisense RNA 1	3.5 FC ↑ (0.0343)				
	mRNA	PSG6	*PSG6*	Pregnancy specific beta-1-glycoprotein 6	3.3 FC ↑ (0.0280)				
	mRNA	C3orf33	*C3orf33*	Chromosome 3 open reading frame 33	3.2 FC ↑ (0.0475)				
	mRNA	MDAC1	*TMEM190*	Transmembrane protein 190	2.4 FC ↑ (0.0275)				
	mRNA	COL8A1	*COL8A1*	Collagen type VII alpha 1 chain	2.2 FC ↑ (0.0488)				
	mRNA	LTF	*LTF*	Lactotransferrin	1.9 FC ↑ (0.0340)				
	mRNA	SULT1E1	*SULT1E1*	Sulfotransferase family 1E member 1	1.8 FC ↑ (0.0410)				
	mRNA	TBX15	*TBX15*	T-box transcription factor 15	1.7 FC ↑ (0.0340)				
	mRNA	ATP12A	*ATP12A*	ATPase H+/K+ transporting non-gastric alpha2 subunit	−5.1 FC ↓ (0.0340)				
	mRNA	LOC401233	*/*		−3.8 FC ↓ (0.0000)				
	mRNA	CLDN4	*CLDN4*	Claudin 4	−3.7 FC ↓ (0.0488)				
	mRNA	LOC643338	*C15orf62*	Chromosome 15 open reading frame 62	−3.2 FC ↓ (0.0450)				
	mRNA	SCGB2A2	*SCGB2A2*	Secretoglobin family 2A member 2	−3.1 FC ↓ (0.0233)				
	mRNA	TCN1	*TCN1*	Transcobalamin 1	−2.6 FC ↓ (0.0220)				
	mRNA	GPR78	*GPR78*	G protein-coupled receptor 78	−1.7 ↓ (0.0175)				
	mRNA	CACNA1E	*CACNA1E*	Calcium voltage-gated channel subunit alpha1 E	−1.6 FC ↓ (0.0471)				
	mRNA	CYP3A7	*CYP3A7*	Cytochrome P450 family 3 subfamily A member 7	−1.2 FC ↓ (0.0433)				

Legend: LH+7 = the seven day after luteinising hormone (LH) surge. TVUS = transvaginal ultrasound. MRI = magnetic resonance imaging. CA125 = cancer antigen 125 biomarker. MS = the mid-secretory phase of the menstrual cycle. FC = a fold change. H-score = a method of assessing staining intensity in immunohistochemistry. miRNA = micro RNA. mRNA = messenger RNA. lncRNA = long-noncoding RNA. * pfp = statistical values presented as percentage of false positives..

**Table 2:** Gene lists associated with endometrial receptivity in women with adenomyosis or endometriosis and healthy women. Altered endometrial expression levels of genes and proteins in women with adenomyosis and endometriosis during the window of receptivity and biomarkers of uterine receptivity were retrieved from the literature survey. The gene nomenclature of extracted data was edited according to the HUGO Gene Nomenclature Committee (HGNC) database.

Group of Women	Gene List
Adenomyosis	*AKR1B10, ATP12A, ATP1A2, C15orf62, C3orf33, CACNA1E, CDKN3, CHD5, CLDN4, COL11A1, COL8A1, CYP3A7, ERCC6L, FNDC1, GPR78, HOXA10, IL10, ITGB3, KCNA4, KLF12, LIF, LIFR, LIPH, LTF, MB, MIR21, MMP20, NR4A1, PSG6, SCGB2A2, SPC25, SPINK2, SPP1, SST, STAT3, SULT1E1, TBX15, TCN1, TMEM190, TMPRSS11B, TUBAL3, ZNF295-AS1.*
Endometriosis	*ABCB11, ABCC3, ACKR1, ACO2, ADGRF1, AFF4, AGT, AIMP1, ALPI, AMY1A, AMY2A, AMY2B, ANXA2, ANXA5, AOC1, ATF3, BST2, C1QA, C1QTNF6, CA1, CA12, CASP5, CCBE1, CCL3, CCL3L1, CCL3L3, CCL8, CCN1, CCT8, CDA, CDK5R1, CELF1, COL12A1, CORO1B, CRABP1, CRISP3, CST7, CTSW, CWH43, CXCL2, CYP3A5, DDIT4L, DDX17, DEPP1, DLG5, DNAJC3, DST, EDNRB, EGR1, EGR2, EGR3, EIF1, EIF4A1, EIF4A2, ENPP3, FMN2, FOS, FOSB, GALP, GSN, GUCY1B1, GZMA, HACD1, HOXA9, HPCAL4, HSP90B1, FNA21, IL6, IMMT, JUNB, KCNK2, KRIT1, KRT18, KRT5, KRTAP19-2, LAMA3, LCK, LONRF2, LPP, LRRD1, LTB4R2, LUZP1, MALL, MAP4, MAPK8, MET, MIR135A1, MIR138-1, MIR138-2, MIR1915, MIR194-2, MIR196A1, MIR196A2, MIR219B, MIR22, MIR26B, MIR3196, MIR339, MIR365B, MIR3686, MIR374B, MIR4251, MIR4252, MIR4254, MIR4425, MIR4723, MIR505, MIR542, MIR548AA2, MIR548AP, MIR548T, MIR5585, MIR921, MMP26, MUC7, MYL12A, NCR1, NEAT1, NFAT5, NR4A1, NR4A3, PAX8, PCSK5, PCYOX1, PDHB, PER1, PITX1, PLEK, PLEKHA2, POMZP3, PRDX6, PRIM2, PRRC2C, PTAFR, RAB9BP1, RBBP4, RGS1, RIF1, RIN1, RNF150, RNH1, RSRP1, S100A3, S100A8, SAP30L, SCG2, SCGB2A2, SEMA3C, SERPINB8, SHB, SLA, SLC15A4, SLC1A1, SLC44A2, SMG1, SOCS3, SON, SP3P, TAF6L, TGFB3, THRAP3, TRIM15, TRPM6, TUBA1C, VDAC1P1, VEGFA, VHL, VIM, YBX1, YBX1P2, YWHAE, ZFP36, ZIC2.*
Healthy	*ABCC3, ACADSB, ADAMTS1, ALPL, AMIGO2, ANG, ANO1, ANXA2, ANXA4, AOX1, APOD, ARG2, ARID5B, ASPM, ATP1B1, ATP6V0E2, ATP6V1A, BARD1, BCL6, BUB1B, C1R, C4BPA, CAPN6, CATSPERB, CCNB2, CDA, CDC20, CDK1, CENPE, CEP55, CFD, CLDN4, CLU, COL16A1, COMP, CP, CRABP2, CSRP2, CTNNA2, CXCL13, CXCL14, DDX52, DEFB1, DEPP1, DEPTOR, DKK1, DLGAP5, DPP4, DYNLT3, ECI2, ECM1, EDN3, EDNRB, EFNA1, ENPEP, EPHB3, FANCI, FOSL2, FXYD2, G0S2, GABARAPL1, GADD45A, GALNT12, GALNT4, GAS1, GAST, GBP2, GDF15, GPX3, GREM2, HABP2, HEY2, HLA-DOB, HPSE, ID4, IDO1, IL15, IMPA2, KCNG1, KIF11, KIF20A, KIF4A, KMO, KRT7, LAMB3, LIF, LMCD1, LMOD1, LRRC17, LYPD3, MAOA, MAP2K6, MFAP2, MFAP5, MMP26, MPPED2, MSX1, MT1G, MT1H, MT2A, MTCL1, NDC80, NDRG1, NDRG2, NNMT, NRG2, OLFM1, OLFM4, PAEP, PAQR4, PBK, PENK, PLA1A, PLAAT3, PLAAT4, PMEPA1, POLD4, POSTN, PRC1, PRKCQ, PRR15L, PRUNE2, PTPRR, RASSF2, RETREG1, RNASE4, RPRM, S100A1, S100A4, S100P, SCGB2A2, SERPINA5, SERPING1, SFRP4, SLC1A1, SNX10, SOD2, SORD, SOX17, SPDEF, SPP1, SYNE2, TACC3, TAGLN, TBC1D2, TCN1, THBD, TMSB15A, TOP2A, TRH, TSPAN8.*

**Table 3:** Retrieved overlapping enriched pathways after gene set enrichment analysis (GSEA) between adenomyosis and endometriosis gene lists. Interleukin-4 and interleukin-13 signalling (marked in bold) served as a source for prioritisation of annotated genes for validation experiment.

		Adenomyosis				Endometriosis			
Reactome Pathway ID	Number of Total Reactions	Annotated Genes	Reaction Count	*p*-Value	FDR-Value	Annotated Genes	Reaction Count	*p*-Value	FDR-Value
R-HSA-8950505: Gene and protein expression by JAK-STAT signalling after Interleukin-12 stimulation	36	*IL10*	1	0.043	0.197	*CA1*, *ANXA2*	2	0.043	0.450
R-HSA-6783783: Interleukin-10 signalling	15	*IL10, STAT3*, *LIF*	15	6.26 × 10^−4^	5.38 × 10^−2^	*IL6*, *CCL3L1*, *CCL3L3*, *PTAFR*, *CCL3*, *CXCL2*	2	6.08 × 10^−7^	2.35 × 10^−4^
R-HSA-6785807: **Interleukin-4 and interleukin-13 signalling**	46	*IL10*, *STAT3*, *LIF*	19	2.60 × 10^−3^	0.086	*SOCS3*, *IL6*, *VIM*, *FOS*, *JUNB*, *VEGFA*, *HSP90B1*	5	1.37 × 10^−4^ E-04	0.015
R-HSA-6798695: Neutrophil degranulation	10	*TCN1*, *LIPH*, *CHD5*, *CACNA1E*, *MMP20*, *LTF*	7	0.020	0.197	*CDA*, *AOC1*, *GSN*, *ANXA2*, *SLC44A2*, *CRISP3*, *PTAFR*, *CXCL2*, *PRDX6*, *CCT8*, *DNAJC3*, *BST2, SLC15A4*, *SCG2*, *KCNK2, S100A8*	9	0.014	0.360
R-HSA-8849474: PTK6 activation of STAT3	9	*STAT3*	6	0.031	0.197	*SOCS3*	3	0.007	0.281

**Table 4:** Comparison of study groups. Values reported as median (95% confidence interval (CI) for median).

Clinical Characteristics	Adenomyosis Group (*n* = 9)	Control Group (*n* = 13)	*p*-Value *
Age (years)	33 [32; 39]	36 [32; 39]	0.946
BMI (kg/m^2^)	29.4 [17.8; 34.6]	22.8 [19.6; 24.0]	0.057
Endometrial thickness (mm)	7.3 [4.6; 9.7]	9.2 [7.4; 13.0]	0.030
Number of previous ART	2 [1; 3]	2 [1; 4]	0.387
Female sterility:			0.522
primary	5	9	
secondary	4	4	

* Values of statistical significance were obtained by Mann–Whitney U test.

**Table 5:** The fold change of selected genes in study groups. Values reported as median (95% CI for median).

Candidate Gene	Adenomyosis Group (*n* = 9)	Control Group (*n* = 13)	*p*-Value
*IL6*	0.664 [0.402; 1.589]	1.054 [0.533; 2.224]	0.333
*LIF*	0.761 [0.140; 4.317]	2.717 [0.423; 6.870]	0.262
*IL10*	0.716 [0.295; 2.080]	1.081 [0.523; 1.995]	0.193
*SOCS3*	0.597 [0.223; 2.780]	1.446 [0.935; 3.283]	0.301
*JUNB*	1.106 [0.419; 1.557]	1.242 [0.714; 1.953]	0.526
*FOS*	0.844 [0.420; 1.657]	1.120 [0.509; 1.705]	0.271

*p*-values were calculated by non-parametric Mann–Whitney U test.

## Supplementary Materials

The following are available online at https://www.mdpi.com/2218-273X/10/9/1311/s1, Figure S1: Clustering of the PPIN which was constructed from adenomyosis, endometriosis and healthy gene lists using Cytoscape/STRING App, Table S1: Complete results of GSEA applied in clusters 1, 2 and 3 following PPIN clustering., Table S2: Complete results of GSEA applied in Reactome tool for overlapping pathways between adenomyosis and endometriosis gene lists.
