# Supplementary material for: Determining the Molecular Background of Endometrial Receptivity in Adenomyosis
Source: Biomolecules. 2020 Sep 11;10(9):1311. doi: 10.3390/biom10091311 (PMC7563201; doi:10.3390/biom10091311)

Construction of PPIN from adenomyosis, endometriosis and healthy gene lists using STRING

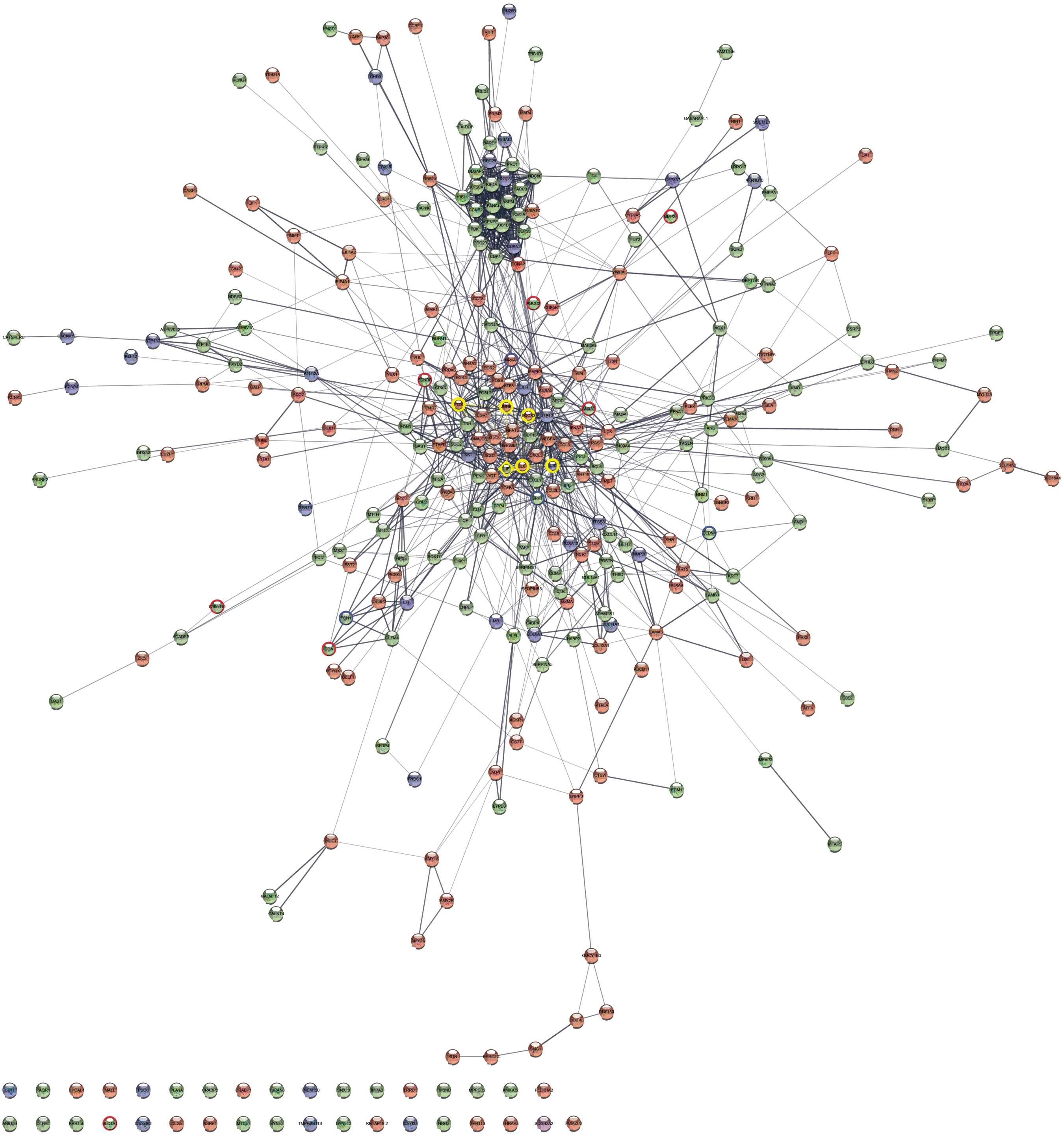

Edge-weighted Spring Embedded Layout with the attribute "score" (316 nodes, 1130 edges)

MLC clustering;

- Granularity parameter: 2.5
- Array source: the overall STRING confidence score

LEGEND:

Node Fill Color	STATUS
(blue)	Adenomyosis gene list
(red)	Endometriosis gene list
(green)	Healthy gene list

Green nodes mapped to healthy gene list that are encircled with blue or red refer to a shared genes with adenomyosis and endometriosis gene list, respectively. Shared SCGB2A2 node between adenomyosis, endometriosis and healthy genes is marked with violet color. Yellow encircled nodes and edges refer to prioritized candidate genes used in validation experiment.

Obtained clusters

Cluster 1 (46 nodes, 210 edges)

Cluster 2 (26 nodes, 225 edges)

Cluster 3 (11 nodes, 12 edges)

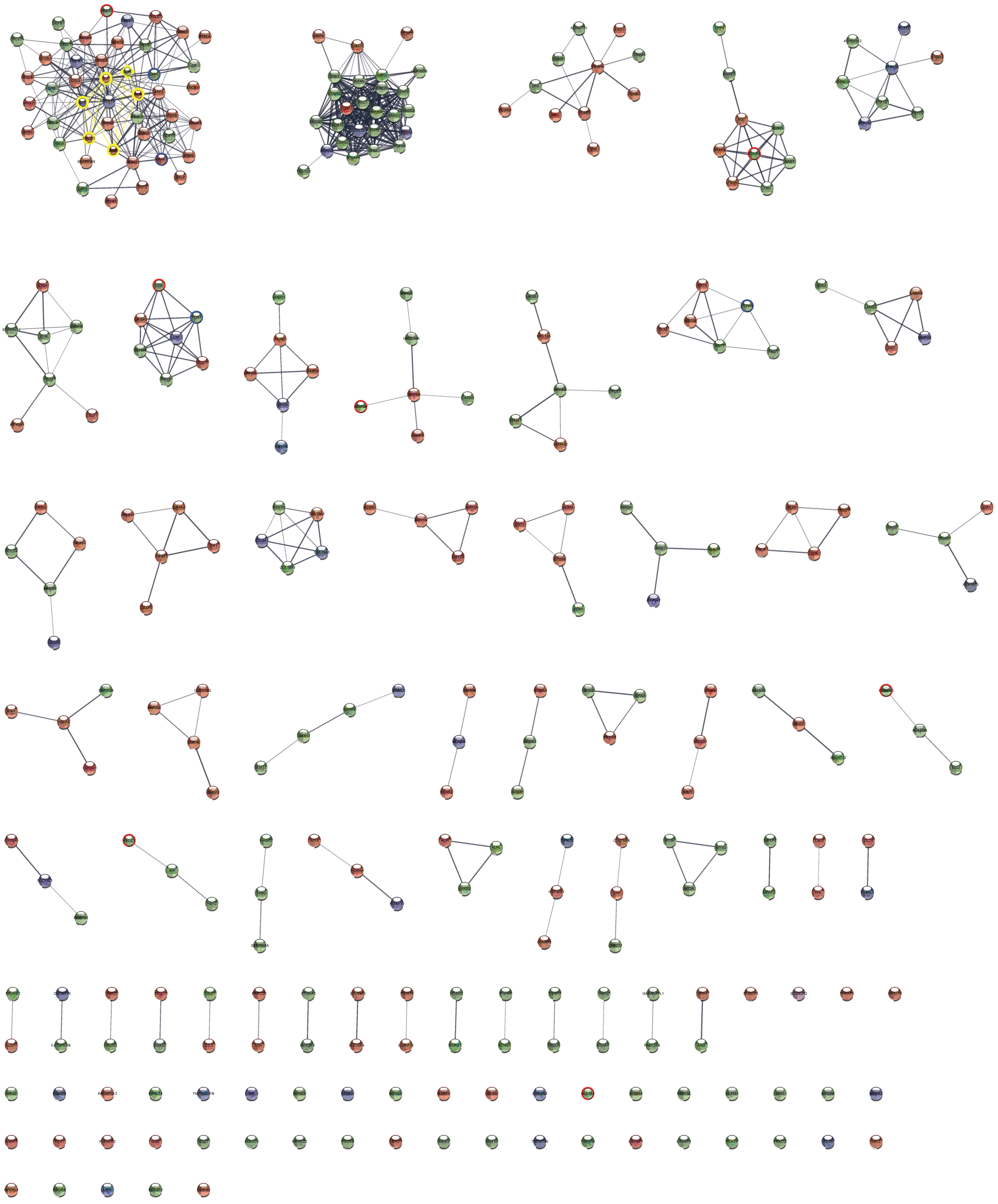

Zoom of cluster 1, 2 and 3

Cluster 1
(46 nodes, 210 edges)

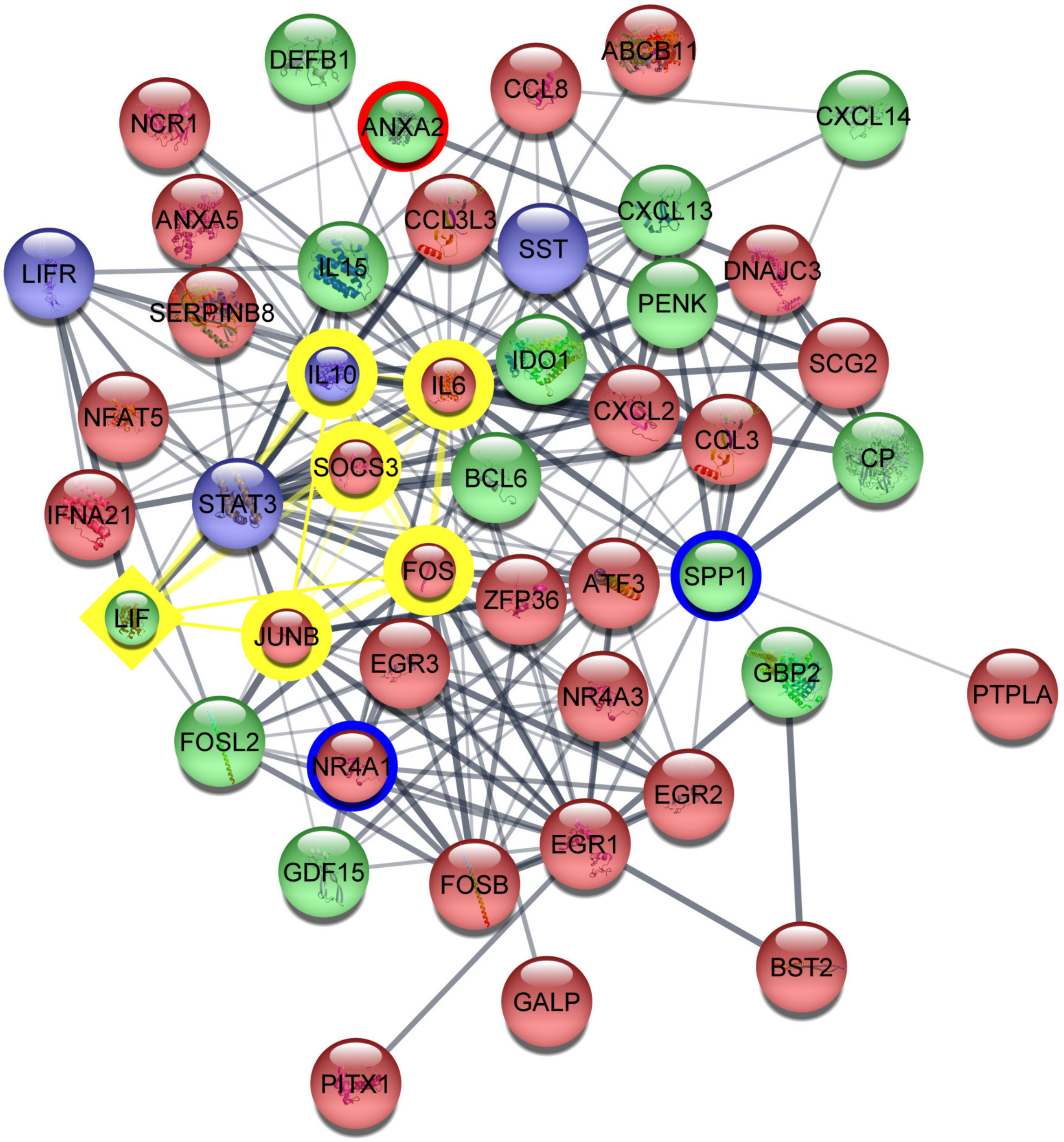

Cluster 2
(26 nodes, 225 edges)

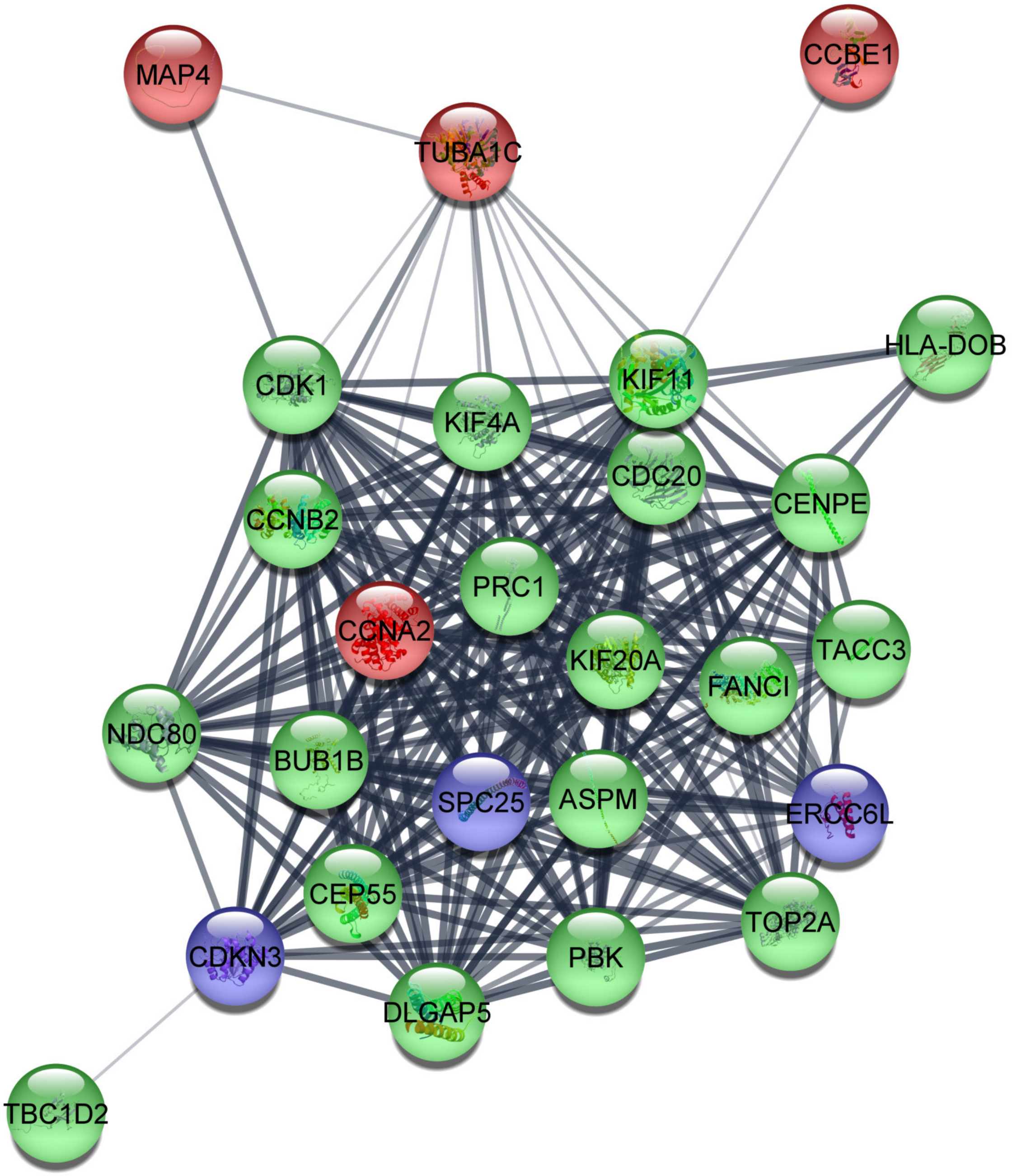

Cluster 3
(11 nodes, 12 edges)

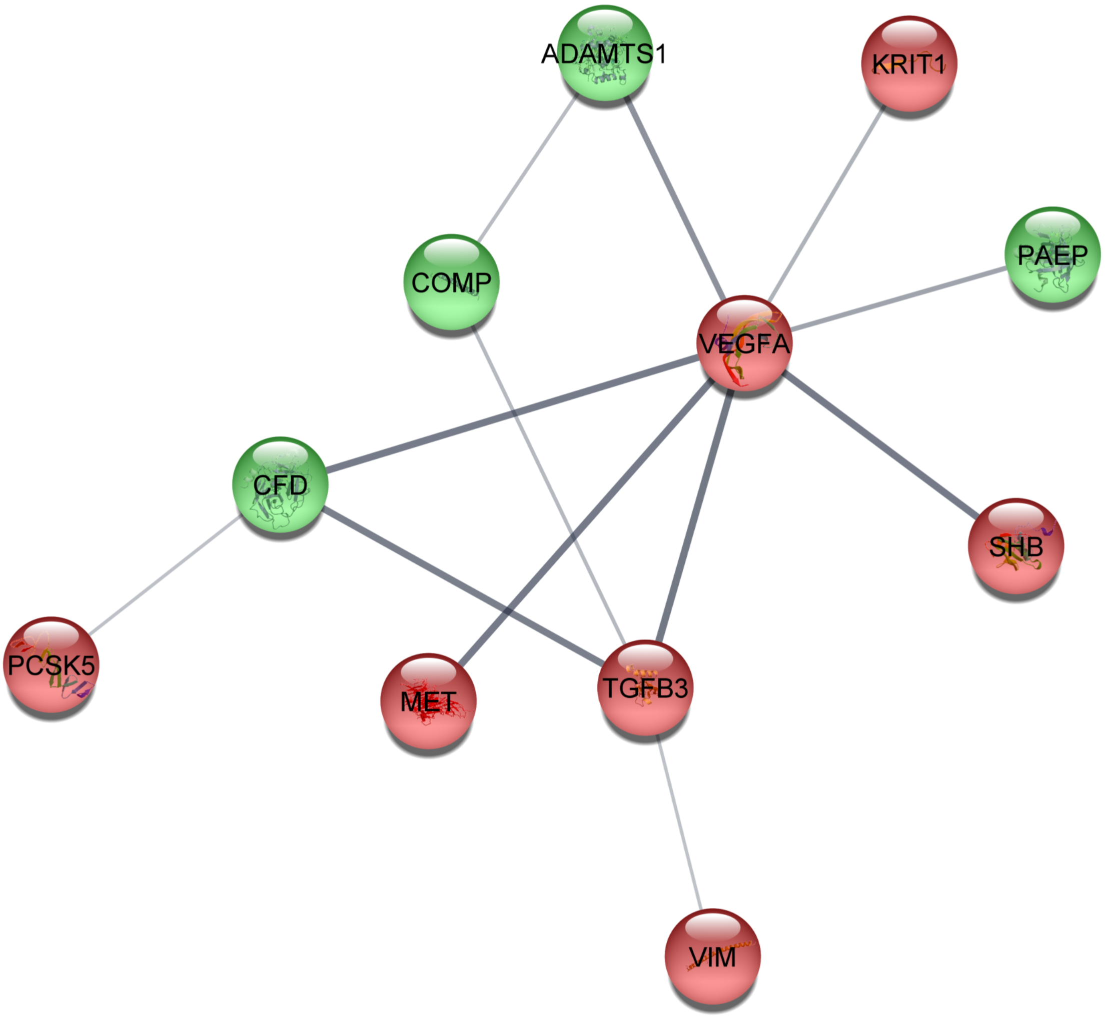

Supplement: Supplementary file 1 [file biomolecules-10-01311-s001.zip › Supplementary Figure S1_ Workflow of PPIN clustering.pdf]
